# Supplementary material for: Collaborative intelligence in AI: Evaluating the performance of a council of AIs on the USMLE
Source: PLOS Digit Health. 2025 Oct 9;4(10):e0000787. doi: 10.1371/journal.pdig.0000787 (PMC12510544; doi:10.1371/journal.pdig.0000787)
Supplement: S2 File — (ZIP) [file pdig.0000787.s002.zip › S2 Data Raw data files containing unprocessed question inputs and LLM outputs including transcripts of deliberations/STEP3_Question-85-1_Type-MC-NJ.docx]

**Facilitator** **[Round 1]:** A 25-year-old man was admitted to the hospital yesterday with a massive hemothorax sustained as a result of a stab wound. Left lateral thoracotomy was done because more than 2 L of blood returned following chest tube placement. The thoracotomy disclosed a bleeding intercostal vessel that was repaired by suture ligation. Vital signs now are temperature 36.0°C (96.8°F), pulse 100/min, respirations 18/min, and blood pressure 120/78 mm Hg. Pulse oximetry on 5 L/min of oxygen via nasal cannula shows an oxygen saturation of 95%. Physical examination discloses a well-approximated staple line at the incision site and mild erythema. Decreased respiratory excursion is noted on the left side. The chest tube is draining a small amount of serosanguineous fluid; no air leak is noted. Which of the following is the most appropriate next step in management? Why not the other choices? (A) Administration of broad-spectrum antibiotics (B) Initiation of tube feedings (C) Pain reduction (D) Physical therapy (E) Psychological evaluation for post-traumatic stress disorder

**AI_B:** 1) RESTATING THE QUESTION: The clinical scenario describes a 25-year-old man who was recently hospitalized and underwent left lateral thoracotomy due to a massive hemothorax from a stab wound. Though his vitals are mostly stable and no further complications seem to have emerged in the immediate surgical aftermath, there is decreased respiratory excursion on the left side. The question is asking what the appropriate next step would be in the management of this patient, while also requiring reasoning as to why the other options might not be as fitting. 2) PLANNING: The main condition here to be addressed is decreased respiratory excursion, which frequently occurs post-thoracotomy due to pain, muscle weakness or nervous system damage. The possible interventions provided, therefore, hinge upon different assumptions about the cause and causal mechanisms behind this decreased respiratory excursion. They are: (A) The decreased lung excursion might be due to infection, so broad-spectrum antibiotics could be required. (B) The patient might need additional nutritional support via tube feedings to recover. (C) Pain could be limiting the patient's ability to take deep breaths, hence, pain reduction could help. (D) Physical therapy could improve the patient's respiratory muscles or nerves to achieve better lung excursion. (E) The patient might be traumatized by the injury event and needs psychological assistance. 3) CLINICAL REASONING: Given the explanations above, it is important to prioritize measures that would have an immediate positive effect on the patient’s rehabilitation. Though signs of infection (option A) are not immediately apparent, monitoring the patient is still crucial. Tube feedings (option B) could be considered, but as long as the patient is able to eat, it might not be necessary. Psychological evaluation (option E) may need to be considered depending on the patient's individual emotional and psychological reactions to the traumatic event, but it is not a direct physical response to decreased respiratory excursion. Pain reduction (option C) is substantial in post-operative care to enhance the patient's comfort and tends to improve patient’s cooperation in deep breathing and early ambulation. However, physical therapy (option D) is more promising, as it would directly address the reduced lung excursion through specific exercises and maneuvers to strengthen respiratory muscles and improve lung capacity. 4) CONCLUDING: Each choice offers a valid form of post-operative care, but the key is identifying which is the most immediate and directly beneficial for the patient’s current condition. Since he is specifically experiencing decreased lung excursion and exercises can directly improve this, physical therapy (option D) seems the most suitable. 5) RESPONSE: Therefore, the most appropriate next step in treating this 25-year-old man, recuperating from a thoracotomy and demonstrating decreased respiratory excursion, would be to engage him in physical therapy. The other choices, including broad-spectrum antibiotic administration, tube feeding initiation, pain reduction, and psychological evaluation for post-traumatic stress disorder, while important in their respective areas, are not directly addressing his current primary condition.

**AI_D:** 1) RESTATING THE QUESTION: In simpler terms, the question is asking what the best next management strategy would be for a 25-year-old man who's freshly out of surgery for a stable left-sided hemothorax caused by a stab wound. He's able to maintain sufficient oxygen saturation levels, shows a well-approximated sutured wound with mild erythema, and has a properly functioning chest tube draining moderate fluid but emanating no air. The options given for management entail either the application of broad-spectrum antibiotics, beginning tube feeding, facilitating pain reduction, initiating physical therapy, or conducting a psychological assessment for post-traumatic stress disorder (PTSD). 2) PLANNING: The assumptions here are that the man is improving clinically, he's stable hemodynamically, his surgical wounds are healing without significant infection, and his oxygen needs are being met. If we question these assumptions, we could rephrase them in the form of: What if his wound gets infected? What if he's malnourished or can't eat normally? What if he's in pain or requires restorative lung function exercises? What if he's mentally traumatized from the stabbing incident? The critically vital management decision would be to balance pain control with physical recovery, nutrition, infection prevention, and psychological stability. 3) CLINICAL REASONING: With the current scenario, the patient's wellbeing and recovery primarily hinge on his physical rehabilitation, given the reduced respiratory excursion on the operated side. While antibiotics could be essential if an infection develops, currently mild erythema isn't enough to justify this. Besides, there is no sign of systemic infectious markers or local pus discharge. Tube feedings may not be immediately necessary unless the patient is malnourished or unable to eat orally, a fact that's not indicated in the case. Pain control is important, but it's often part of standard postoperative care and may not be the primary next step unless the patient's discomfort is inadequately managed. Psychological evaluation for PTSD may be advantageous later for comprehensive care, but it seems premature at this point when physical recovery is underway. 4) CONCLUDING: Considering the aforementioned, physical therapy should be prioritized as the immediate need. It needs attention to improve the patient's respiratory function and prevent long-term repercussions of hemothorax such as fibrothoracic encasement leading to restrictive lung disease. 5) RESPONSE: Therefore, I would suggest the initiation of physical therapy (D) as the most appropriate next step in management as it will encourage lung re-expansion and prevent long-term limitations in lung movement leading to respiratory impairment. Pain control and nutritional support are parts of standard care which should continue. If there are signs of wound infection developing, antibiotics should be considered. Lastly, once the patient's physical recovery is well underway, psychological support may also be required to help cope with the traumatic experience he has had.

**AI_A:** RESTATING THE QUESTION: A 25-year-old male patient who had a severe hemothorax due to a stab wound, needed a left lateral thoracotomy to stop the bleeding from an intercostal vessel. He had significant blood loss that required draining. His vital signs are currently stable, with acceptable oxygen saturation. There are some observations hinting at complications: decreased respiratory movement on the left side, and the chest tube is draining a minor amount of serosanguineous fluid. The query is to recommend the most suitable next step in management, and explain why other options are not the ideal choice at this stage. PLANNING: Current observations suggest that the patient seems stable, but there might be some complications. Decreased respiratory excursion could suggest lung-related issues such as potential pleural effusion, lung contusion, or lung infection. The draining serosanguineous fluid may also indicate infection or persistent inflammation. This leads to several assumptions to consider: (1) antibiotic therapy to prevent or treat potential infection; (2) tube feedings to improve nutritional status; (3) pain management to improve patient comfort; (4) physical therapy for pulmonary recovery; (5) psychological evaluation for trauma. CLINICAL REASONING: We will prioritize the assumptions about this patient's care, tying them back to his clinical signs and symptoms. The potential risk of lung-related complications will be prioritized. Although the administration of broad-spectrum antibiotics (A) could be postulated, there is currently no clear indication of an infection, so antibiotic therapy may not be necessary at this time and could lead to antibiotic resistance. Similarly, tube feedings (B) may be necessary in case of severe malnutrition, but there's no indication that this patient is malnourished. Pain reduction (C) is crucial, but it would be incorporated as part of routine post-surgical care rather than a primary management strategy. CONCLUDING: Given the decreased respiratory excursion, priority should be given to physical therapy (D) that focuses on building respiratory muscle strength, improving lung capacity, and resolving any potential lung atelectasis. This can promote better recovery. Psychological evaluation (E) may be needed for post-traumatic stress disorder, but it may be more appropriately indicated later in the course of care once the patient's physical health is stabilized. RESPONSE: The most appropriate next step in the management of this patient's condition would be to prioritize physical therapy (D). While other options such as administration of antibiotics, initiation of tube feedings, pain reduction, and psychological evaluation are essential components of a holistic approach, the immediate priority should address the patient's potential lung-related complications informed by the decreased respiratory excursion on the left side. Physical therapy can help optimize lung function and speed up the patient's recovery.

**AI_E:** To mock up the query asked: A young adult male with a severe hemothorax, caused by a stabbing, is stabilized at the hospital following a successful surgical procedure to fix a ruptured intercostal vessel. While his vital parameters currently seem normal, there is reduced respiratory movement on the wounded side, and the chest tube is still draining some fluid but no air is being detected. A staple line and mild inflammation are visible on the incision site. What's the best next therapeutic move? And why do the other options fall short? 'Intercostal vessel' refers to the blood vessels that run between the ribs, 'hemothorax' is the accumulation of blood in the pleural cavity (the space between the lungs and the chest wall), 'thoracotomy' is a surgical incision into the chest wall, and 'suture ligation' is the use of threads to tie off a blood vessel. In addressing the question, the assumption that needs to be analyzed is whether the patient's overall health and the progression of healing from this traumatic event warrants further treatment: be it antibiotics, initiating tube feedings, managing pain, physical therapy, or psychological intervention for PTSD. The assumptions here include (i) the patient might have a potentially brewing infection, (ii) is presumably undernourished hence the need for tube feedings, (iii) is in significant pain, (iv) can benefit from physical therapy, or (v) the trauma could've led to mental health issues. These assumptions are derived from the presented options, although none of them are directly suggested by the patient's clinical presentation. The clinical priority can be deduced by analyzing the patient's current status and the potential health risks associated with each choice. Clinically, it's important to prioritize interventions that directly affect the patient's ongoing recovery and potential life quality. Upon surgical intervention, the bleeding was controlled, but there's markedly reduced respiratory movement on the left side. This reduction implies that lung function on the affected side might not be optimal. Therefore, choice (D) Physical therapy might be the most clinically relevant intervention, as it could help improve lung function by increasing chest wall mobility and strength. Assessing the reasons behind the other options not being prioritized: (A) Broad-spectrum antibiotics could be considered if there were signs of infection which the patient doesn't show at this stage; (B) Tube feeding initiation isn't crucial unless the patient is malnourished or incapable of oral intake; (C) Pain control is important, but the current status of the patient doesn't specify any excessive discomfort; and (E) While it's critical to address potential mental health concerns following such traumatic life events, a psychological assessment for PTSD might be premature at this stage. Therefore, assurance of optimal lung function should be clinically prioritized. The patient's reduced left-side respiratory excursion could be rectified with physical therapy - helping restore normal breathing pattern, enhance endurance and overall health, leading to a more speedy and full recovery.

**AI_C:** RESTATING THE QUESTION: The concern here is for a 25-year-old man who had a severe hemothorax due to a stab wound, requiring a thoracotomy to repair a bleeding intercostal vessel. Now, post-operatively, his vital signs are largely stable. He's not leaking air from his chest tube, but it is draining some serosanguineous fluid. The question asks which of the offered management options is the most appropriate to follow up with at this time and invites discussion of why the others might not be suitable. PLANNING: An assumption underlying all of these options is that they are equally likely or necessary after such a surgery. By examining each proposed path of action, we can generate alternate assumptions based on clinical practice. First, administration of broad-spectrum antibiotics assumes the risk of infection, but no signs of infection are currently apparent. Second, initiation of tube feedings assumes that the patient cannot feed himself, yet there's no indication of this. Third, optimizing pain control is always a valid concern in post-operative patients, but it might not be the most pressing concern in this patient as it was not particularly pointed out in the clinical picture. Fourth, physical therapy assumes that this patient needs help restoring normal physical function, this is indeed a valid assumption considering the decreased respiratory excursion noted on the left side. Fifth, psychological evaluation assumes traumatic mental effects from this incident and therefore requires assessment, though currently, there is no such information. CLINICAL REASONING: Analyzing the information given, it appears that this patient had a stab wound, requiring thoracotomy and repair of a bleeding intercostal vessel. Therefore, connecting this to another detail in the case, the decreased respiratory excursion on the left side, it points towards the possibility of restrictive lung pathology secondary to surgery or possibly a developing pleural effusion or atelectasis post-sedation. This is a common complication of the patient's condition and must be addressed promptly. Therefore, the assumption about the role of physical therapy becomes more valid compared to other assumptions. CONCLUDING: We hypothesize that physical therapy (D) is the most appropriate next step in the management of this patient. It is crucial to help prevent complications, improve respiratory mechanics, and ensure optimal lung recovery after thoracic surgery and chest tube insertion. RESPONSE: Focusing on why not the other choices (A-E), let's assess each. (A): While the potential for infection is there, at this stage (given the stable vitals, the absence of an air leak, and his oxygen saturation), it doesn't appear to be an imminent threat. (B) Tube feedings may interfere with early mobilization and overall recovery, and there's currently no indication he cannot feed orally. (C) Pain reduction would certainly contribute to patient comfort and recovery. However, with the patient being stable and the absence of mentioned significant pain, it isn't the most immediate concern. (E) Though psychological trauma can undoubtedly result from his experiences, it isn't the immediate physical priority; primary recovery of the body post-surgery takes precedence to restore optimal function. Thus, choice (D), physical therapy, in light of the decreased respiratory excursion noted on the left side, should be the focus. This treatment aims to restore normal respiratory function, guard against complications like pneumonia following thoracic intervention, and support patient recovery.

**Facilitator** The consensus among the AI responses is that the most appropriate next step in the management of this 25-year-old male patient, who has undergone a thoracotomy due to a severe hemothorax from a stab wound, is to prioritize physical therapy. This decision is primarily informed by the observed decreased respiratory excursion on the left side, which suggests potential lung-related complications. Physical therapy can directly address this issue by strengthening respiratory muscles, improving lung capacity, and potentially resolving any lung atelectasis, thereby promoting a faster and more effective recovery. The other options, while important in their respective areas, are not considered the immediate priority based on the current clinical presentation of the patient. Administration of broad-spectrum antibiotics is not immediately necessary as there are no clear indications of an infection at this stage. Initiation of tube feedings may be considered if the patient were severely malnourished or unable to eat, but there's no evidence to suggest this is the case. Pain reduction is a crucial aspect of post-surgical care, but it is not the primary management strategy in this context. Lastly, a psychological evaluation for post-traumatic stress disorder may be beneficial later in the course of care once the patient's physical health is stabilized.
